# Activation of the RARα Attenuated CSF Hypersecretion to Inhibit Hydrocephalus Development via Regulating the MAFB/MSR1 Pathway

**DOI:** 10.3390/ijms24032586

**Published:** 2023-01-30

**Authors:** Hanhai Zeng, Kaibo Yu, Junyou Wang, Jingya Ye, Huaijun Chen, Chaoran Xu, Ting Chen, Feng Yan, Gao Chen, Chi Gu

**Affiliations:** 1 Department of Neurological Surgery, The Second Affiliated Hospital, Zhejiang University School of Medicine, Hangzhou 310009, China; 2 Key Laboratory of Precise Treatment and Clinical Translational Research of Neurological Diseases, Zhejiang University, Hangzhou 310003, China; 3 School of Medicine, Zhejiang University, Hangzhou 310030, China

**Keywords:** hydrocephalus, CSF, RARα, MSR1, spontaneous hypertensive rat

## Abstract

Hydrocephalus has been observed in rats with spontaneous hypertension (SHRs). It has been demonstrated that activation of the oxidative stress related protein retinoic acid receptor alpha (RARα) has neuroprotective impacts. Our investigation aims to determine the potential role and mechanism of RARα in hydrocephalus. The RARα-specific agonist (Am80) and RARα inhibitor (AGN196996) were used to investigate the role of RARα in cerebrospinal fluid (CSF) secretion in the choroid plexus of SHRs. Evaluations of CSF secretion, ventricular volume, Western blotting, and immunofluorescent staining were performed. Hydrocephalus and CSF hypersecretion were identified in SHRs but not in Wistar–Kyoto rats, occurring at the age of 7 weeks. The RARα/MAFB/MSR1 pathway was also activated in SHRs. Therapy with Am80 beginning in week 5 decreased CSF hypersecretion, hydrocephalus development, and pathological changes in choroid plexus alterations by week 7. AGN196996 abolished the effect of Am80. In conclusion, activation of the RARα attenuated CSF hypersecretion to inhibit hydrocephalus development via regulating the MAFB/MSR1 pathway. RARα may act as a possible therapeutic target for hydrocephalus.

## 1. Introduction

Hydrocephalus frequently develops following intracranial hemorrhage, resulting in poor clinical outcomes [1,2]. Invasive cerebrospinal fluid (CSF) shunting, an empirical ‘one-size-fits-all’ strategy with significant morbidity because of frequent shunt blockages and infections necessitating surgical revision, is still the preferred therapy for hydrocephalus. Hydrocephalus individuals of all ages have an urgent unmet necessity for targeted therapeutic approaches [3]. In 1986, researchers showed increasing ventricular dilation in spontaneously hypertensive rats (SHRs) at 4 to 8 weeks of age [4]. The mechanisms of such spontaneous hydrocephalus have not been explained, but they may provide insight into the mechanisms of adult hydrocephalus, such as those caused by intracranial hemorrhage and traumatic brain injury. This model may also be an easy and reproducible model for exploring potential mechanisms.

Hydrocephalus is a common CSF physiology disorder that results in abnormal enlargement of the cerebral ventricles [5,6,7]. Excessive CSF production or too little drainage and absorption may cause hydrocephalus. It is commonly acknowledged that hydrocephalus is caused by an initial reduction in CSF reabsorption due to restriction of intraventricular CSF flow and/or malfunction of extra-ventricular arachnoid granulations, namely the accumulation of CSF inside the intraventricular as a result of dysfunctional homeostasis pathways [8]. Previous studies focused more on the obstruction or limited absorption of the cerebrospinal fluid outflow tract and less on the problem of excessive cerebrospinal fluid production [8,9]. Karimy et al. proposed and verified the key role of excessive cerebrospinal fluid production in the development of hydrocephalus, and until 2017, attention began to be paid to relevant research gradually [10]. However, the breakthroughs that can be made are still limited [11,12], mainly because most studies use IVH or ICH-IVH models, and hydrocephalus is regarded as a complication after intracranial hemorrhage. However, hydrocephalus occurs not only after intracranial hemorrhage, but also after normal intracranial pressure hydrocephalus, infant hydrocephalus, and post-traumatic hydrocephalus, and the related pathophysiological mechanisms are different [13,14,15]. Therefore, we try to explore the potential mechanism of hydrocephalus from SHRs rats and look for possible therapeutic targets, so as to provide some reference for subsequent research. Based on the above two reasons, in this study, we mainly focus on the possible mechanism of hydrocephalus caused by cerebrospinal fluid hypersecretion in addition to using SHRs as a hydrocephalus model animal.

Retinoic acid receptors (RARs) are members of the nuclear receptor superfamily, and RARs are important oxidative stress-related proteins, which are regulated by the oxidative stress state of the body. However, studies on other mechanisms of activation of RARs are relatively limited. Studies have shown that RARs regulate cell differentiation, embryonic development, and metabolism [16]. In addition, RARs are expressed in the adult central nervous system (CNS) [17]. Early studies have shown that the activation of RARs protects the brain from inflammation-related damage in Alzheimer’s disease and stroke [18,19]. Am80, named Tamibarotene, a selective RARα agonist, is thought to have a significant inhibitory effect on inflammation in CNS diseases [20,21]. Inflammation is strongly associated with the development of hydrocephalus [22], suggesting that RARα-targeted therapy may be beneficial in hydrocephalus. However, the involvement of Am80 in hydrocephalus in anti-neuroinflammation and CSF secretion has not been demonstrated.

Macrophage scavenger receptor 1 (MSR1) is a heterogeneous molecular found on the surface of phagocytes [23]. Recent findings suggest that MSR1 may regulate nuclear factor kappa (NFK) B pathways via binding with several ligands [24]. As an upstream regulatory transcription factor of MSR1, it is reported that V-maf musculoaponeurotic fibrosarcoma oncogene homolog B (MAFB) mediates the expression of MSR1 to reduce inflammatory damage in CNS disease [23]. The activation of RARα could enhance MAFB expression and accelerate the resolution of cerebral post-ischemic inflammation [23]. Therefore, we postulated that RARα receptor stimulation decreased CSF secretion via regulating inflammation in hydrocephalus by promoting the MAFB/MSR1 pathway.

## 2. Results

### 2.1. SHRs Experienced Hydrocephalus and CSF Hypersecretion in Week 7

**Figure 1:**
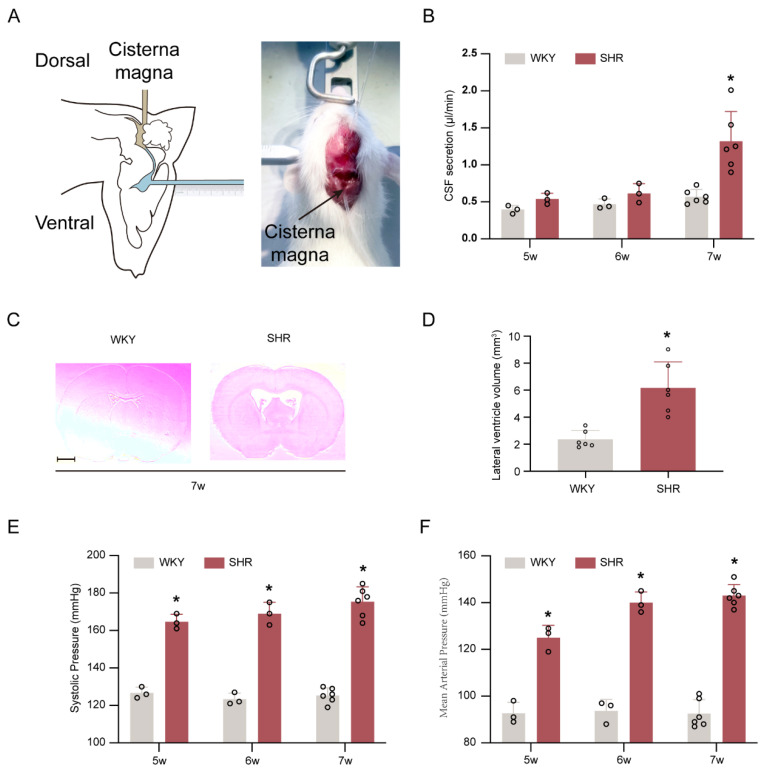
CSF hypersecretion and hydrocephalus occurs in SHRs at seven weeks of age. (**A**) Left, schematic of the method used for in vivo quantification of CSF secretion in rats. Right, direct measurement of CSF secretion in a live rat. (**B**) Quantification of the rate of CSF secretion at weeks 5 (*n* = 3), 6 (*n* = 3), and 7 (*n* = 6). * *p* < 0.05 versus WKY rats at week 7. (**C**) Representative photomicrographs of coronal sections of rat brains (at −0.6 mm from the bregma) depicting ventricular volume at week 7 in SHRs compared with age-matched WKY rats. Scale bar, 2 mm. (**D**) Quantification of lateral ventricle volume in SHRs (*n* = 6) and WKY rats (*n* = 6) at weeks 7. * *p* < 0.05 versus WKY rats at week 7. (**E**,**F**) SHRs had significantly higher systolic blood pressure and mean arterial pressure than WKY rats at weeks 5 (*n* = 3), 6 (*n* = 3), and 7 (*n* = 6). * *p* < 0.05 versus WKY group. Values are means ± SD.

We assessed the time point of CSF secretion by quantifying CSF production rates in WKY and SHR groups at weeks five, six, and seven. At weeks five and six, CSF production rates were equal in WKY rats and SHRs. However, at week seven, SHRs exhibited obvious CSF hypersecretion (*p* < 0.05, Figure 1A,B). Quantification of ventricular volume at week seven also demonstrated a substantial variation among rats in SHR groups (*p* < 0.05, Figure 1C,D). At weeks five, six and seven, the systolic blood pressure and mean arterial pressure of WKY rats were significantly lower compared to those of SHRs (*p* < 0.05, Figure 1E,F). Thus, the development of CSF hypersecretion and hydrocephalus in the SHR happens after the onset of hypertension.

### 2.2. Temporal Expression of RARα, MAFB, and MSR1

**Figure 2:**
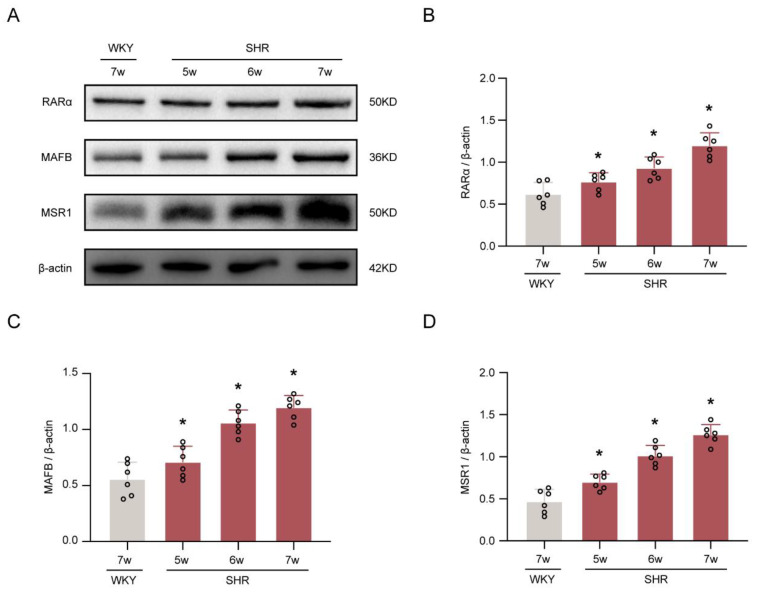
Temporal expression of RARα, MAFB, and MSR1 in SHRs. (**A**) Representative Western blotting images of RARα, MAFB, and MSR1. (**B**–**D**) Quantitative analyses of RARα, MAFB, and MSR1. *n* = 6. * *p* < 0.05 versus WKY rats at week 7. Values are means ± SD.

The Western blotting results determined the temporal expression of RARα, MAFB, and MSR1 in the choroid plexus of the ventricle (Figure 2A). In SHRs, the levels of RARα, MAFB, and MSR1 gradually upregulated and reached a peak at week 7 among weeks 5, 6, and 7 (*p* < 0.05, Figure 2B–D).

### 2.3. Stimulation of RARα-Inhibited CSF Hypersecretion and Ameliorated Hydrocephalus

**Figure 3:**
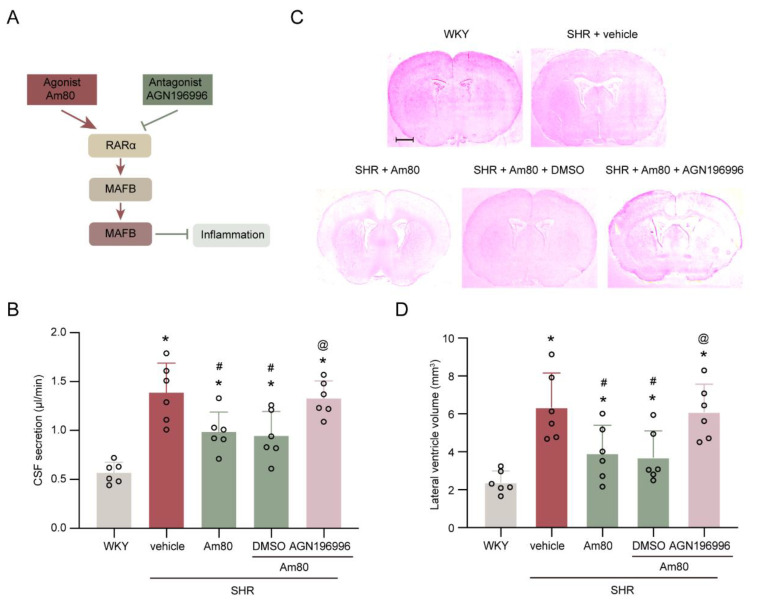
AGN196996 abolished the protective effects of Am80. (**A**) Schematic diagram of the experimental design. (**B**) Quantification of the rate of CSF secretion at week 7 (*n* = 6). (**C**) Representative photomicrographs of coronal sections of rat brains (at −0.6 mm from the bregma), depicting ventricular volume at week 7. Scale bar, 2 mm. (**D**) Quantification of lateral ventricle volume at week 7 (*n* = 6). * *p* < 0.05 versus WKY rats at week 7, # *p* < 0.05 vs. SHR + vehicle group, @ *p* < 0.05 vs. SHR + Am80 + DMSO group. Values are means ± SD.

The effectiveness of Am80 on CSF hypersecretion and hydrocephalus in SHRs was examined. Compared with the WKY group, rats in the SHR + vehicle group demonstrated a significantly increased CSF production at week 7 and ventricular volume at week 7 (*p* < 0.05, Figure 3B–D). Moreover, with the treatment for two weeks, Am80 conferred a protective effect on excess CSF production and ventricular volume to attenuate the impairments that happened in SHRs at week 7, compared to the vehicle-treated SHRs (*p* < 0.05, Figure 3B–D).

### 2.4. Stimulation of RARα Decreased the Number of Microglia/Macrophage

**Figure 4:**
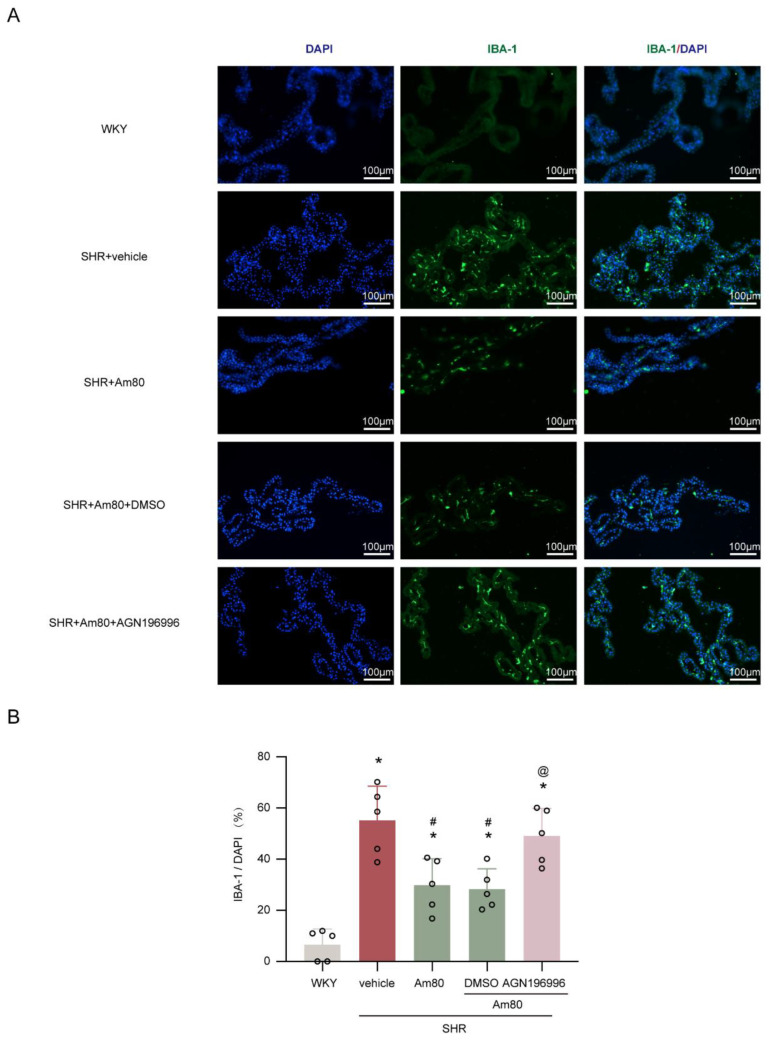
AGN196996 abolished the effects of Am80 on microglia/macrophage activation. (**A**) Examples of choroid plexus Iba-1 positive cells in rats at week 7. (**B**) Quantification of Iba-1 positive cells at week 7 (*n* = 5). Scale bar, 100 μm. * *p* < 0.05 versus WKY rats at week 7, # *p* < 0.05 vs. SHR + vehicle group, @ *p* < 0.05 vs. SHR + Am80 + DMSO group. Values are means ± SD.

Given the essential role of inflammation in hydrocephalus, the next part of this study was conducted to determine whether the protective effects of Am80 in the pathological process following hydrocephalus were exerted by mediating the inflammatory response. We confirmed a significant increase of Iba-1 positive cells in SHRs (*p* < 0.05, Figure 4A,B), whereas the SHR + Am80 group had a lower number of Iba-1 positive cells (*p* < 0.05, Figure 4A,B).

### 2.5. Inhibition of RARα Reversed the Impacts of Am80

To block the RARα, the selected antagonist AGN196996 was utilized. Following Am80 and AGN196996 injections, the CSF hypersecretion and ventricular volume of SHRs were significantly increased (*p* < 0.05, Figure 3B–D), relative to the SHR + Am80 + DMSO group. In the same circumstances, the Iba-1 positive cells were also increased substantially (*p* < 0.05, Figure 4A,B).

### 2.6. Am80 Stimulated RARα to Reduce CSF Hypersecretion via the MAFB/MSR1 Pathway

**Figure 5:**
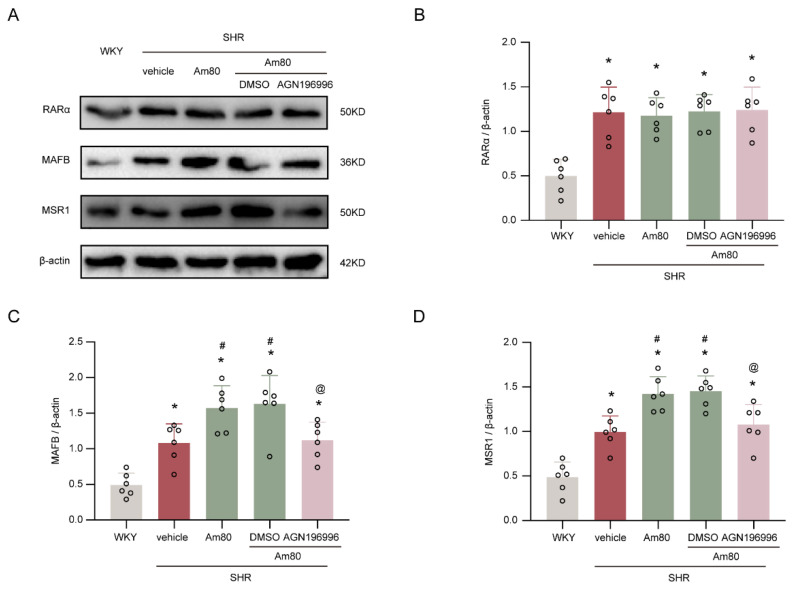
Am80 stimulated RARα to reduce CSF hypersecretion via the MAFB/MSR1 pathway. (**A**) Representative Western blotting images of RARα, MAFB, and MSR1 at week 7. (**B**–**D**) Quantitative analyses of RARα, MAFB, and MSR1 at week 7 (*n* = 6). * *p* < 0.05 versus WKY rats at week 7, # *p* < 0.05 vs. SHR + vehicle group, @ *p* < 0.05 vs. SHR + Am80 + DMSO group. Values are means ± SD.

The MAFB and MSR1 expressions were significantly elevated in SHRs post-treatment with Am80, but there was no alteration in the RARα level (*p* < 0.05, Figure 5A–D). Compared with the SHR + Am80 + DMSO group, the group treated with RARα inhibitor AGN196996 abolished the effect caused by Am80 on the levels of MAFB and MSR1 (*p* < 0.05, Figure 5A–D).

## 3. Discussion

In the current investigation, we showed that the CSF secretion in SHRs was notably elevated at seven weeks of age, and the onset of hydrocephalus may be correlated with CSF hypersecretion. Upregulation of RARα decreased the secretion of CSF, thus alleviating the hydrocephalus, whereas the inhibition of RARα reversed the above protective properties. Importantly, the mechanism of RARα on hydrocephalus was associated with MAFB, MSR1, and neuroinflammation. Moreover, inhibiting RARα abolished the favorable impacts of Am80 on the upregulation of MAFB, MSR1, and the inhibition of inflammation. Taken together, the current outcomes demonstrated that activation of the RARα attenuated CSF hypersecretion to inhibit hydrocephalus development, at least in part through the MAFB/MSR1 pathway.

Previous research has shown that SHRs have increasing ventricular dilation that persists over time [4,25,26]. However, in a subsequent investigation, an angiotensin-converting enzyme inhibitor lowered blood pressure in SHRs but did not attenuate ventriculomegaly [27]. In addition, MRI verified the presence of ventriculomegaly in SHRs at the age of ninety days [25]. The recent research showed the SHR’s potential as a model for spontaneous hydrocephalus [5]. In the present investigation, SHRs developed CSF hypersecretion by week seven but not at weeks five or six. Therefore, we took SHRs to investigate hydrocephalus. In 2017, Karimy et al. reported a role of CSF hypersecretion in the pathogenesis of hydrocephalus that had not been acknowledged before [10]. Then, continuous reports have revealed and verified this mechanism, but the relevant research is still limited [11,12]. This is the first time that CSF hypersecretion is explored in SHRs.

The neuroinflammation was reported to stimulate CSF secretion [11,12]. Inflammation in CSF and in the choroid plexus tissue may represent the underlying pathology of hydrocephalus [28]. Since the 1840s, physicians have observed inflammation in the brain and cerebrospinal fluid space in posthemorrhagic hydrocephalus (PHH) and post-infectious hydrocephalus [22]. Restorative inflammation is an important protective response that eliminates foreign organisms, damaged cells, and physical irritants; however, inappropriately triggered or persistent inflammation can initiate or propagate disease, respectively. Recent data have begun to reveal the molecular mechanisms by which inflammation contributes to the pathogenesis of hydrocephalus, including cytokines, immune cells, and signaling pathways. Therapeutic approaches targeting inflammatory mediators may address the drivers of choroid plexus CSF hypersecretion [22]. We identified RARα as a role in regulating macrophage/microglia infiltration in choroid plexus after hydrocephalus. It is documented that the stimulation of RARα decreases the transcription of inflammatory cytokines to inhibit the inflammatory response of lung tissue [29]. In renal disorder, stimulation of RARα inhibits proliferation and inflammation by inducing podocyte differentiation [30]. Additionally, RARα is regarded as a possible therapeutic target for neurodegenerative disorders [31]. In animal models of Alzheimer’s disease, RARα agonists have been demonstrated to decrease congenital neuroinflammation, enhance phagocytosis, and decrease neuropathology [32,33,34,35]. The above recent studies and our study illustrated the role of RARα in inflammation and hydrocephalus.

Am80 has been licensed for the management of acute promyelocytic leukemia as an agonist of RARα [36]. Recent research indicates that Am80 has a crucial function in illnesses in CNS, including ischemic stroke, hemorrhagic stroke, cancer, etc. [20,21,33,37]. However, previous studies have not studied the therapeutic effect of Am80 in hydrocephalus, and the particular protective mechanism following RARα stimulation remains unknown. Our outcomes showed that Am80 substantially inhibited the high secretion of cerebrospinal fluid in hydrocephalus and inhibited the infiltration and activation of macrophages/microglia. At the same time, in vivo experimental results also confirmed that Am80 could reduce the development of hydrocephalus. In general, these findings showed that Am80 could attenuate the occurrence and development of hydrocephalus. Furthermore, Am80 is able to cross the blood–brain barrier (BBB) easily, allowing it to be taken intravenously or orally, making it appropriate for other medical transformation purposes [38].

MAFB, which is strongly expressed in monocytes and macrophages, is a pivotal transcription factor for macrophage differentiation [39,40]. Previous reports have described the effect of MAFB on the regression of inflammation, and deeply participated in the control of aseptic inflammation by promoting the clearance of DAMP [23]. As a component of the scavenger receptor family, MSR1 is mostly expressed by microglia in the central nervous system and scavenges amyloid, aged cell debris, and different cell-damaging compounds [41]. MSR1 can also increase M2 polarization of macrophages for osteogenic differentiation following fracture by stimulating the PI3K Akt pathway [42]. MAFB has been shown to increase the expression of MSR1 and decrease the release of inflammatory cytokines via endocytosis [23]. In this study, we confirmed that RARα might affect the occurrence and development of hydrocephalus through MSR1-mediated neuroinflammation. These results suggest that MAFB/MSR1-mediated macrophage/microglia infiltration and activation are involved in the regulation of cerebrospinal fluid hypersecretion in the occurrence and development of hydrocephalus.

Nevertheless, there are still limits in our findings that require additional in-depth investigation. Firstly, we did not investigate the function of direct activation or inhibition of MSR1 in hydrocephalus. Secondly, although we have shown that RARα activation can have a certain effect on cerebrospinal fluid hypersecretion through MAFB/MSR1, to a certain extent, it does not exclude the effects of other pathways and explain the pleiotropic and unspecific functions. Thirdly, the research of RARα in clinical practice needs further investigation.

## 4. Materials and Methods

### 4.1. Animals

The Ethics Board of the Second Affiliated Hospital, Zhejiang University School of Medicine, authorized all animal experiments (2019-575). All procedures were performed according to the Guide for the Care and Use of Laboratory Animals of the National Institutes of Health. SHRs and age-matched Wistar–Kyoto (WKY) rats (Beijing Vital River Laboratory Animal Technology Co., Ltd., Beijing, China) were maintained in a 12/12 h light/dark cycle environment with controlled humidity and temperature. All rats had a starting body weight of ninety–one-hundred grams. Blood pressure (BP) was determined utilizing the tail cuff technique (CODA 6; Kent Scientific Corporation, Torrington, CT, USA). At weeks 5, 6, and 7, systolic BP (SBP) and mean arterial pressure (MAP) were assessed and documented.

### 4.2. Study Design

All rats were assigned into two experiments. First, 4-week-old SHRs and WKY rats were housed, and their CSF production was measured at weeks 5, 6, and 7 to determine the rates of CSF production [43]. At week 7, ventricle volumes were analyzed by serial section [10]. Western blotting was utilized to quantify RARα, MAFB, and MSR1 levels in the choroid plexus of lateral ventricle from SHRs (weeks 5, 6, and 7) and WKY (week 7) rats. Second, SHRs (week 5) were treated with vehicle, Am80 (Tamibarotene, MCE, HY-14652), and/or DMSO (MCE, HY-Y0320), or AGN196996 (MCE, HY-16682), and WKY rats were used as a control (Figure 3A). At week 7, CSF secretion and the ventricle volume of rats were assessed. Moreover, the choroid plexus of the lateral ventricle was taken for Western blotting and the brain was taken for immunofluorescence.

### 4.3. Am80 Administration

Starting from week 5, Am80 was administered intraperitoneally (IP) every day at a dose of five milligrams per kilogram in SHRs [18]. The duration of treatment was two weeks. SHRs in the control group were injected IP with 0.5% carboxymethyl cellulose (Merck, CAS9000-11-7) solution. Adverse reactions such as diarrhea and weight loss in animals were monitored.

### 4.4. Quantification of CSF Production Rates (Figure 1A)

Following the early reported Karimy’s technique [43], rates of CSF production were determined. In brief, anesthetized animals were placed on a stereotactic device, and a 1.3 mm burr hole (1.7 mm lateral to the bregma, 0.8 mm posterior to the bregma) was created above the left lateral ventricle. After that, the head of the rat was turned 90° to make its nose face downward. The suboccipital tissue were cut down to the cisterna magna to reveal the atlantooccipital ligament. A hole was made in the ligament, and a flexible 23-gauge catheter (PE-20) was moved 5 mm via the foramen of Magendie to the fourth ventricle. The aqueduct of Slyvius was blocked by placing 100 μL of sterile molecular-grade mineral oil (100 μL; Sigma-Aldrich, Taufkirchen, Germany) into the fourth ventricle; this created a closed space for CSF circulation. Keeping the animal in the same posture, a capillary glass tube (CV8010-300; borosilicate; OD, 1 mm; ID, 0.8 mm; length, 30 cm; VitroCom, Mountain Lakes, NJ, USA) was inserted via the burr hole and into the lateral ventricle. At a specific time-point, the CSF volume (V) was computed as V (mm^3^) = π·r^2^·d, in which r is the capillary tube’s radius and d is the distance that the CSF traveled in the capillary. The CSF production rate (μL/min) might be estimated from the slope of the volume–time curve.

### 4.5. Ventricular Volume Evaluation

The brains were removed and kept in formalin (Beyotime Biotechnology, Shanghai, China) for one day at 4 °C. The samples were immersed in 30% sucrose until sinking to the bottom. To eliminate distortion caused by cryo-sectioning and slide mounting, we captured high-resolution images of serial coronal slices of the brain (200 μm apart, 14 levels) while it was placed in the cryostat, utilizing standard criteria for camera location, magnification, and external illumination. ImageJ was utilized to count the number of pixels in the area of the lateral ventricle in every thick slice. Pixels were turned into area in mm^2^, summed over fourteen levels, and multiplied by the distance of every 200 μm to determine ventricular volume [10].

### 4.6. Western Blotting

Western blotting was conducted as reported earlier [44]. Rats were anesthetized, and intracardiac perfusion was performed using 0.1 mol/L cold PBS (Beyotime Biotechnology, ST447). The choroid plexus of the ventricle was taken for sample extraction. The samples were centrifuged for 15 min (12,000× *g*, 4 °C). The protein concentration was measured using a BCA Protein Assay Kit (Thermo Fisher Scientific, Waltham, MA, USA). Equal amounts (40 g) of protein samples were denatured in loading buffer (Beyotime Biotechnology, P0015, Shanghai, China) and isolated utilizing SDS-PAGE. The protein was transported to a membrane of polyvinylidene fluoride (PVDF). The PVDF membranes were blocked in 5% skim milk (Beyotime Biotechnology, P0216) for 1 h at room temperature. The membrane was treated overnight at 4 °C with primary antibodies: rabbit anti-RARα antibody (1:500, bs-22843R, Bioss, Woburn, MA, USA), rabbit anti-MAFB antibody (1:1000, Abcam, Waltham, MA, USA, ab65953), rabbit anti-MSR1 antibody (1:1000, Abcam, Ab151707), and rabbit anti-β-actin antibody (1:5000, Abcam, ab8227). The membranes were treated with horseradish peroxidase-conjugated secondary antibodies (1:5000, ZSGB-BIO, Beijing, China, ZB-2301) for two hours at room temperature. Membranes were identified with the Immobilon enhanced chemiluminescence (ECL) Ultra Western HRP Substrate reagent kit (EMD Millipore Corporation, Billerica, MA, USA) and photographs were examined using ImageJ software (National Institutes of Health).

### 4.7. Immunofluorescence

Rats were anesthetized and perfused with 0.1 mol/L cold PBS (Beyotime Biotechnology, ST447), followed by 4% paraformaldehyde (PFA) (Beyotime Biotechnology, P0099). The brains were harvested and then fixed with 4% PFA overnight and 30% sucrose until sinking to the bottom at 4 °C. Brain slices (8 μm thickness) were blocked for half an hour at room temperature with immunol staining blocking buffer (Beyotime, P0102) and washed with PBS three times. Sections were treated overnight at 4 °C with the primary antibody: rabbit anti-IBA-1 antibody (1:1000, Abcam, ab178847). At room temperature, sections were treated with a secondary antibody for two hours: Goat anti-Rabbit IgG (H + L) Cross-Adsorbed, Alexa Fluor™ 488 (1:500, Invitrogen, Waltham, MA, USA, A-11008). Finally, the sections were covered with 4′,6-diamidino2-phenylindole (DAPI) (Abcam, ab285390). A fluorescence microscope (Lecia, Mannheim, Germany) was employed to observe the photographs. ImageJ program was used to examine the outcomes. To quantify the Iba-1-positive cells, we selected at least three sections per mouse from similar areas of the choroid plexus and analyzed three fields per section at a magnification of ×200 per section.

### 4.8. Statistical Analysis

All outcomes are displayed as average ± standard deviation (SD) or median with an interquartile range. Using the student’s *t*-test, comparisons between two groups were made for data with a normal distribution. If there were more than two groups being compared, a one-way analysis of variance (ANOVA) was conducted. Variables were evaluated using the Mann–Whitney U test or Kruskal–Wallis test, accompanied by the Dunn post hoc test, for the result that was not regularly distributed. Statistical evaluation was conducted employing GraphPad Prism 8 and SPSS. *p* < 0.05 was indicated as substantially significant.

## 5. Conclusions

Our outcomes revealed that Am80, the agonist of RARα, attenuated the macrophage/microglia infiltration, and inhibited the cerebrospinal fluid hypersecretion of SHRs, possibly by regulating the MAFB/MSR1 pathway. Hence, RARα might be a possible therapeutic target to alleviate hydrocephalus.

## Data Availability

The raw data supporting the conclusions of this article will be made available by the authors, without undue reservation.
